# Determinants for the improved thermostability of a mesophilic family 11 xylanase predicted by computational methods

**DOI:** 10.1186/1754-6834-7-3

**Published:** 2014-01-06

**Authors:** Huimin Zhang, Jianfang Li, Junqing Wang, Yanjun Yang, Minchen Wu

**Affiliations:** 1 State Key Laboratory of Food Science and Technology, School of Food Science and Technology, Jiangnan University, 1800 Lihu Avenue, Wuxi, Jiangsu 214122, China; 2 School of Biotechnology, Jiangnan University, 1800 Lihu Avenue, Wuxi, Jiangsu 214122, China; 3 Wuxi Medical School, Jiangnan University, 1800 Lihu Avenue, Wuxi, Jiangsu 214122, China

**Keywords:** Xylanase, Thermostability, Computational method, N-terminus replacement, Site-directed mutagenesis, Xylooligosaccharides

## Abstract

**Background:**

Xylanases have drawn much attention owing to possessing great potential in various industrial applications. However, the applicability of xylanases, exemplified by the production of bioethanol and xylooligosaccharides (XOSs), was bottlenecked by their low stabilities at higher temperatures. The main purpose of this work was to improve the thermostability of AuXyn11A, a mesophilic glycoside hydrolase (GH) family 11 xylanase from *Aspergillus usamii* E001, by N-terminus replacement.

**Results:**

A hybrid xylanase with high thermostability, named AEXynM, was predicted by computational methods, and constructed by substituting the N-terminal 33 amino acids of AuXyn11A with the corresponding 38 ones of *Ev*Xyn11^TS^, a hyperthermostable family 11 xylanase. Two AuXyn11A- and AEXynM-encoding genes, *Auxyn11A* and *AExynM*, were then highly expressed in *Pichia pastoris* GS115, respectively. The specific activities of two recombinant xylanases (reAuXyn11A and reAEXynM) were 10,437 and 9,529 U mg^-1^. The temperature optimum and stability of reAEXynM reached 70 and 75°C, respectively, much higher than those (50 and 45°C) of reAuXyn11A. The melting temperature (*T*_m_) of reAEXynM, measured using the Protein Thermal Shift (PTS) method, increased by 34.0°C as compared with that of reAuXyn11A. Analyzed by HPLC, xylobiose and xylotriose as the major hydrolytic products were excised from corncob xylan by reAEXynM. Additionally, three single mutant genes from *AExynM* (*AExynM*^C5T^, *AExynM*^P9S^, and *AExynM*^H14N^) were constructed by site-directed mutagenesis as designed theoretically, and expressed in *P. pastoris* GS115, respectively. The thermostabilities of three recombinant mutants clearly decreased as compared with that of reAEXynM, which demonstrated that the three amino acids (Cys^5^, Pro^9^, and His^14^) in the replaced N-terminus contributed mainly to the high thermostability of AEXynM.

**Conclusions:**

This work highly enhanced the thermostability of AuXyn11A by N-terminus replacement, and further verified, by site-directed mutagenesis, that Cys^5^, Pro^9^, and His^14^ contributed mainly to the improved thermostability. It will provide an effective strategy for improving the thermostabilities of other enzymes.

## Background

Xylan, the major constituent of hemicellulose, is a heterogeneous polysaccharide with a backbone of β-1,4-D-linked xyloses, and abundantly present in agricultural wastes, such as corncob, wheat bran, and bagasse [1,2]. Because of its heterogeneity and complexity, the complete degradation of xylan is a more complex procedure that requires the synergistic action of several xylanolytic enzymes. Among them, xylanase (endo-β-1,4-D-xylanase, EC 3.2.1.8) is a key enzyme in that it cleaves the internal β-1,4-D-xylosidic linkages of xylan to yield different chain lengths of xylooligosaccharides (XOSs) [3]. Enzymes with xylanolytic activity have been classified into several glycoside hydrolase (GH) families (http:/www.cazy.org/fam/acc_GH.html), whereas most xylanases belong to GH families 10 and 11 [1]. The overall three-dimensional structure of family 11 xylanases consists mainly of one α-helix and two β-sheets which are packed against each other, resembling a partially closed right hand [4].

Recently, it has been increasingly recognized that xylanase plays an important role in lignocellulosic biodegradation. For example, xylanase, used in bioethanol production from lignocellulosic materials, could promote the hydrolysis of cellulose by decomposing xylan which restricts the access of cellulase to the cellulose surface [5]. Unfortunately, most wild-type xylanases are poor in thermostability, which prevented them from being used in bioprocesses where high temperatures were required to improve the availability and solubility of substrates, and to reduce the viscosity and microbial contamination of the reaction solution [6]. Although some thermostable xylanases were isolated from thermophiles, their expression levels and/or specific activities were much lower, making them unable to be used efficiently [7,8]. Therefore, it is desirable to improve the thermostabilities of mesophilic xylanases by genetic engineering. As far as we know, some domains or local regions affecting protein thermostability, such as salt bridge, hydrogen bond, charged surface residue, disulfide bridge, and N- or C-terminus, have been revealed [9,10]. Among those factors, the importance of the N-terminus in maintaining xylanase thermostability was highlighted by some researchers [11,12].

*Ev*Xyn11^TS^ is one of the most thermostable of GH family 11 xylanase as reported previously [13]. In our previous work, a mesophilic family 11 xylanase (AuXyn11A) was isolated from *Aspergillus usamii* E001, having high specific activity, broad pH stability, and strong resistance to metal ions and ethylene diamine tetraacetic acid (EDTA) [14]. Then, the AuXyn11A-encoding gene, *Auxyn11A*, was cloned [15]. The homology alignment, conducted using the DNAMAN 6.0 software (Lynnon, Pointe-Claire, QC, Canada), displayed that the primary structure of AuXyn11A shared 62.4% identity with that of *Ev*Xyn11^TS^, but the identity of N-terminal 38 amino acids (numbered by *Ev*Xyn11^TS^) between them was 36.8%. That, to some extent, implied the significance of the xylanase N-terminus to its thermostability. In this work, the suitable N-terminal region of AuXyn11A to be substituted by the corresponding one of *Ev*Xyn11^TS^ was predicted by computational methods, and a hybrid gene, *AExynM*, was constructed by megaprimer PCR as designed theoretically. Two genes, *Auxyn11A* and *AExynM,* were then expressed in *Pichia pastoris* GS115, respectively. In addition, based on the computational design, three genes, *AExynM*^C5T^, *AExynM*^P9S^, and *AExynM*^H14N^, were obtained by site-directed mutagenesis, and expressed in *P. pastoris*, respectively. The temperature characteristics of reAuXyn11A, reAEXynM, and three recombinant single mutants were analyzed and compared. Finally, the action of reAEXynM or reAuXyn11A on corncob xylan was analyzed by HPLC. To our knowledge, this is the first report on the determinants for the improved thermostability of a mesophilic GH family 11 xylanase predicted by computational methods, as well as experimented by N-terminus replacement and site-directed mutagenesis.

## Results and discussion

### Computational prediction of the hybrid xylanase

**Figure 1:**
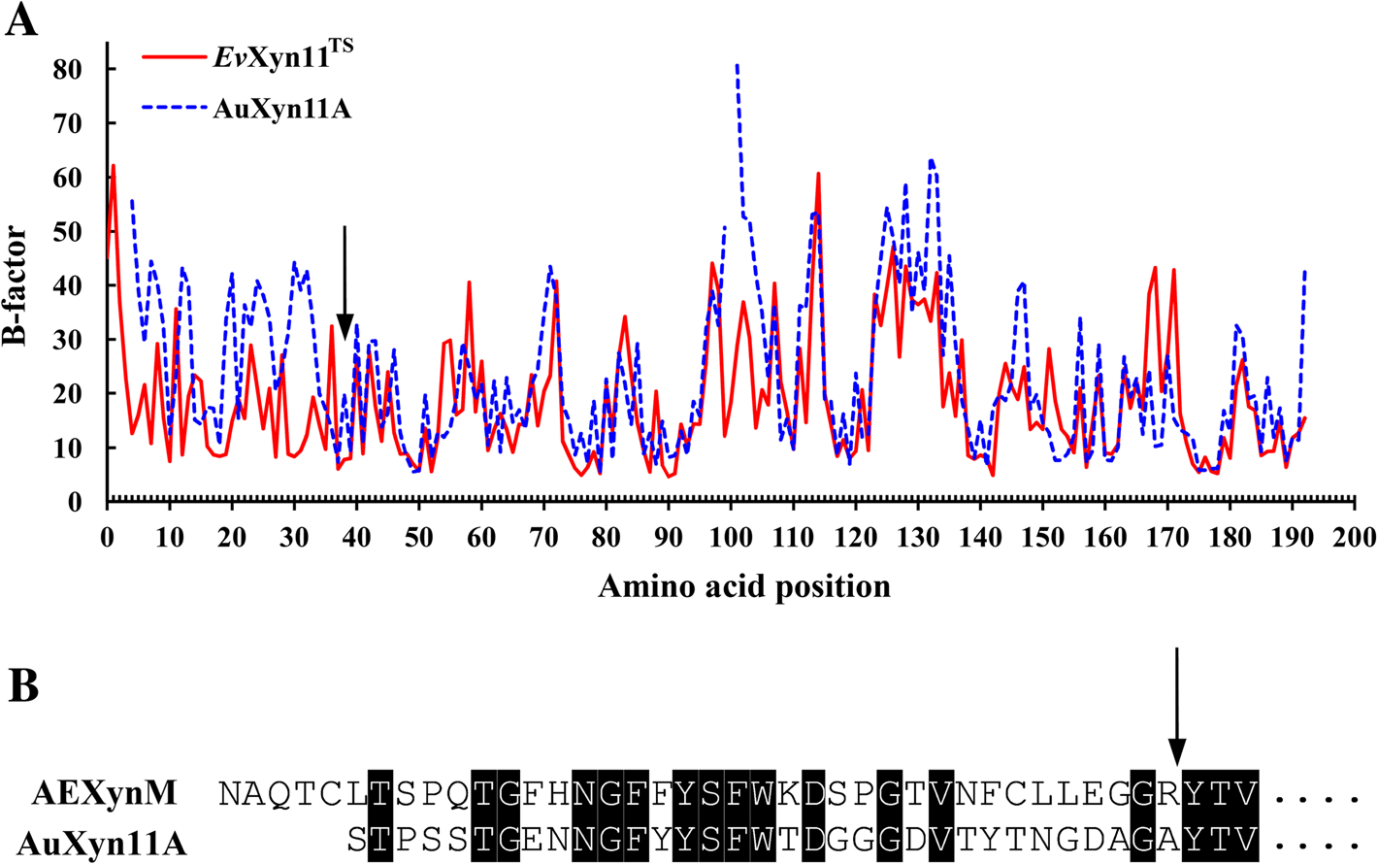
**Computational prediction of AEXynM by comparison of B-factor values. (A)** The B-factor values of amino acid residues of AuXyn11A (dashed line) and *Ev*Xyn11A^TS^ (solid line) were calculated after a 15 ns MD simulation process at a temperature of 300 K. **(B)** The homology alignment of N-terminal sequences between AEXynM and AuXyn11A. The site of N-terminus replacement is marked with a bold arrow. MD, molecular dynamics.

It has been demonstrated that the conformation of mesophilic protein is more flexible than that of the thermophilic one [16]. To quantify the flexibility of protein, the notion of B-factor values was introduced to reflect smearing of atomic electron densities with respect to their equilibrium positions as a result of thermal motion and positional disorder [17]. In this work, the B-factor values of AuXyn11A and *Ev*Xyn11^TS^ were calculated by using the B-FITTER program (Figure 1A). After comparing the B-factor values between them, the N-terminal segment from Ser^1^ to Ala^33^ of AuXyn11A was selected, corresponding to the most pronounced degrees of thermal motion, namely flexibility. As a result, the AEXynM was designed by substituting the N-terminus of AuXyn11A with the corresponding one from Asn^1^ to Arg^38^ of *Ev*Xyn11^TS^ (Figure 1B).

**Figure 2:**
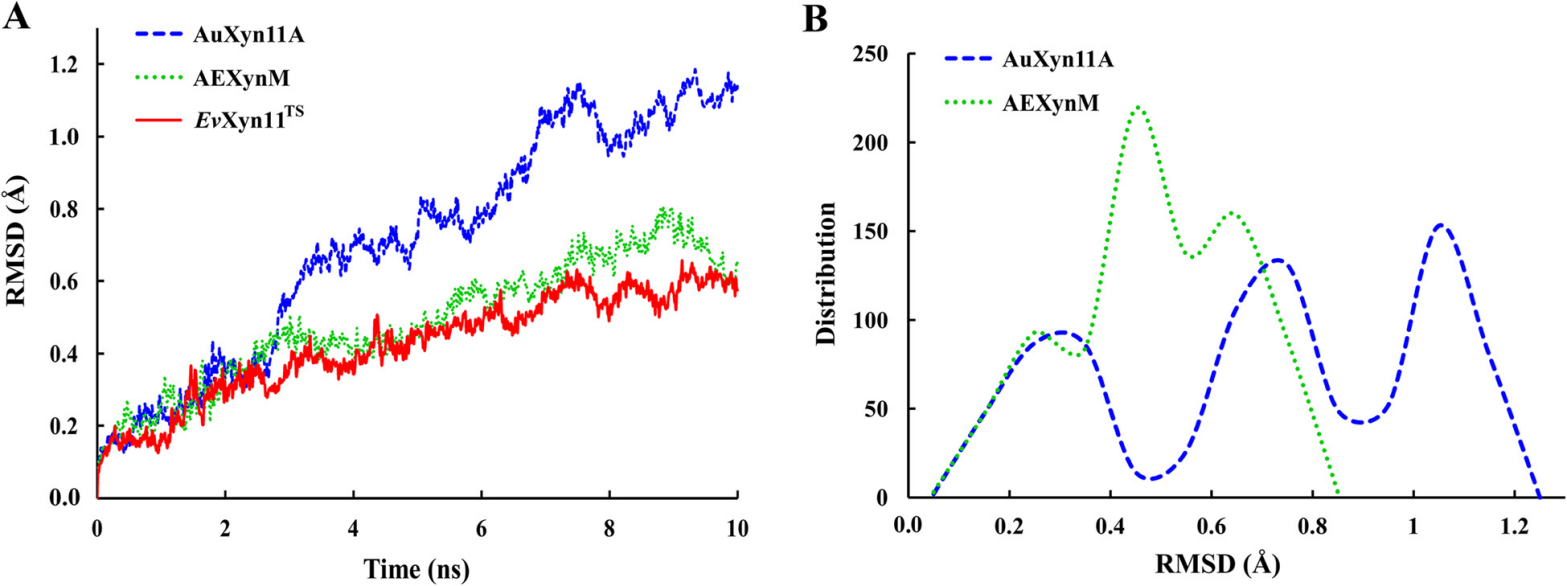
**Calculation and distribution of the RMSD values. (A)** The curves of RMSD values of AuXyn11A (dashed line), AEXynM (dotted line), and *Ev*Xyn11^TS^ (solid line), respectively, after MD simulation processes at 500 K for 10 ns. **(B)** The distributions of RMSD values of AuXyn11A (dashed line) and AEXynM (dotted line), respectively. MD, molecular dynamics; RMSD, root mean square deviation.

The three-dimensional structures of AuXyn11A, AEXynM, and *Ev*Xyn11^TS^ were homologically modeled, and subjected to molecular dynamics (MD) simulation processes, followed by calculating their root mean square deviation (RMSD) values, respectively (Figure 2A). The MD simulation trajectory of AEXynM was almost equal to that of *Ev*Xyn11^TS^. However, the RMSD value of AuXyn11A after equilibration was much larger than that of AEXynM or *Ev*Xyn11^TS^. Simultaneously, the distributions of RMSD values (that is, atomic displacement ranges) of AuXyn11A and AEXynM were statistically analyzed, respectively, using the Origin8 software (Figure 2B). The RMSD values of AuXyn11A were mainly focused on 1.05 Å and those of AEXynM on 0.45 Å, indicating that AEXynM was much more rigid than AuXyn11A. Based on the analytical result that the rigidity of a protein was positively related to its thermostability [18], the designed hybrid xylanase AEXynM was predicted to be more thermostable than the wild-type xylanase AuXyn11A.

### Construction and expression of *AExynM*

An approximate 120 bp band of the DNA fragment SyX was amplified from pUCm-T-*Syxyn11* by first-round PCR with primers SyX-F and SyX-R. Then, using pUCm-T-*Auxyn11A* as the template, a complete hybrid gene *AExynM* of 600 bp was amplified by second-round PCR with primers SyX and AuX-R. The DNA sequencing result verified that the cloned *AExynM* was exactly 596 bp in length (containing *Eco*RI and *Not*I sites), coding for a hybrid xylanase AEXynM of 193 amino acids. The homology alignment of amino acid sequences indicated that the identities of AEXynM with AuXyn11A and *Ev*Xyn11^TS^ were 87.6 and 74.7%, respectively.

The *P. pastoris* transformant that could resist higher concentrations of geneticin G418 might contain multiple copies of integration of a heterologous gene into the *P. pastoris* genome, which could potentially lead to a higher expression level of the heterologous protein as elucidated in the manual of the Multi-Copy Pichia Expression Kit (Invitrogen, Carlsbad, CA, USA). The protein expression level, however, was not directly proportional to the concentration of G418 [19]. Therefore, all *P. pastoris* transformants, resistant to 1.0, 2.0, and 4.0 mg mL^-1^ of G418, were picked out for flask expression tests. After the transformants were induced by adding 1.0% (v/v) methanol at 24-hour intervals for 72 hours, their cultured supernatants were harvested and used for xylanase activity and protein assays, respectively. Among all tested transformants, two recombinant strains, labeled as *P. pastoris* GSAu4-2 and GSAEM4-8, expressing the highest reAuXyn11A and reAEXynM activities of 646.6 and 580.8 U mL^-1^, respectively, were screened. No xylanase activity was detected in the cultured supernatant of the *P. pastoris* GSC under the same expression conditions.

### Purification of reAuXyn11A and reAEXynM

**Figure 3:**
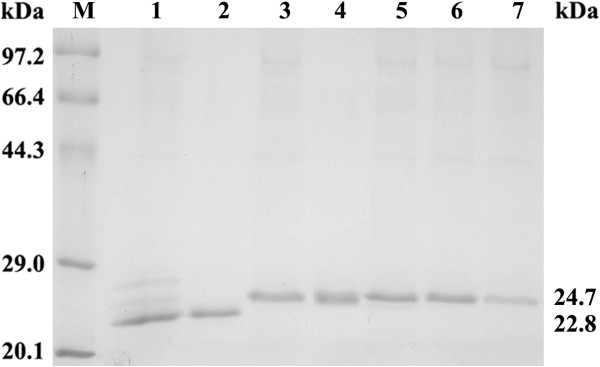
**SDS-PAGE analysis of the recombinant xylanases.** Lane M, standard protein molecular mass markers; lanes 1, 3, 5, 6, and 7, cultured supernatants of *P. pastoris* GSAu4-2, GSAEM4-8, GSAEM^C5T^4-1, GSAEM^P9S^4-3, and GSAEM^H14N^4-5, respectively; and lanes 2 and 4, purified reAuXyn11A and reAEXynM with apparent molecular masses of 22.8 and 24.7 kDa, respectively.

One of the advantages of the *P. pastoris* expression system was that the purities of expressed recombinant proteins were very high according to the description of the Multi-Copy Pichia Expression Kit, which could greatly simplify the purification procedures. It was reported that the purity of the recombinant *A. usamii* xylanase D (reAuXyn11D) expressed in *P. pastoris* was more than 85% [20]. In this work, the amount of expressed reAuXyn11A or reAEXynM, assayed by protein band scanning, accounted for over 82% of that of total protein (Figure 3, lane 1 or 3). Therefore, reAuXyn11A or reAEXynM was purified to homogeneity only by a simple combination of ammonium sulfate precipitation, ultrafiltration, and Sephadex G-75 gel filtration, displaying a single protein band with an apparent molecular mass of 22.8 or 24.7 kDa on SDS-PAGE (Figure 3, lane 2 or 4). The specific activities of reAuXyn11A and reAEXynM, towards 0.5% (w/v) birchwood xylan under the standard assay conditions, were 10,437 and 9,529 U mg^-1^, respectively. The specific activity of reAEXynM was much higher than those of the thermostable xylanase A from *Thermomonospora fusca* expressed in *P. pastoris* (117.3 U mg^-1^) [21] and of commercialized thermostable xylanase 2 (40 U mg^-1^) reported from Sigma-Aldrich (St Louis, MO, USA).

### Temperature characteristics of reAuXyn11A and reAEXynM

**Figure 4:**
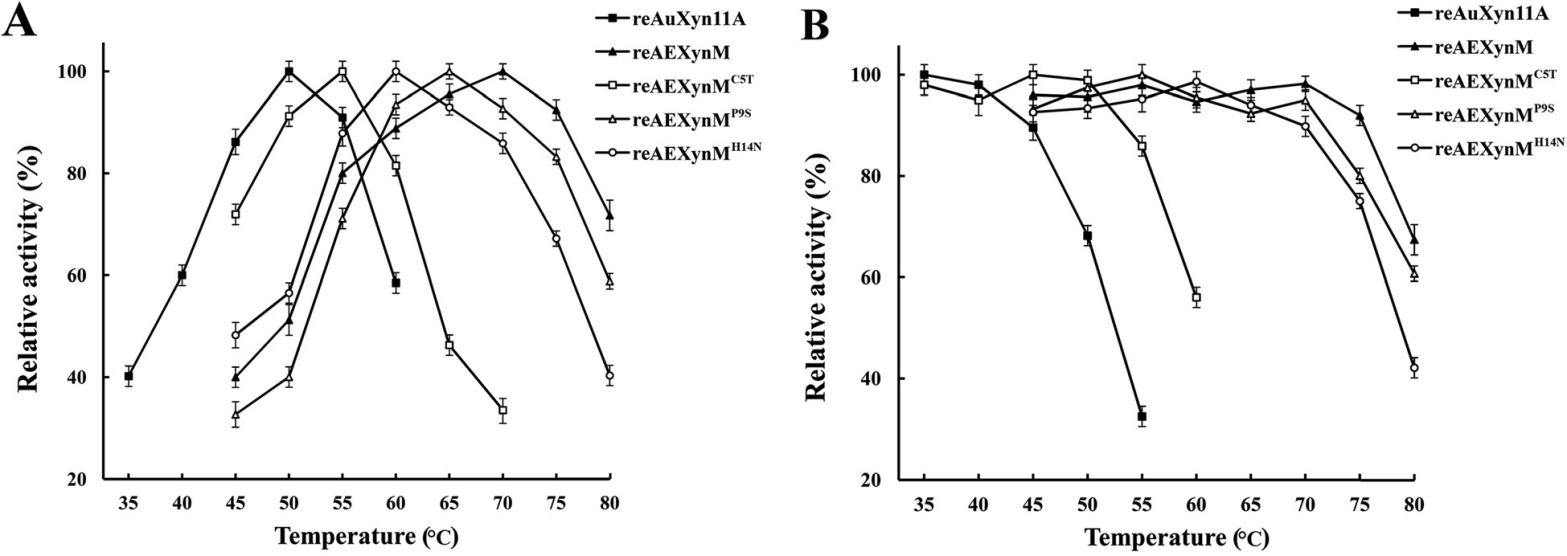
**Temperature optimum and stability of the recombinant xylanases. (A)** The temperature optima were measured under the standard assay conditions, but temperatures ranged from 35 to 60°C for reAuXyn11A as well as from 45 to 80°C for reAEXynM and its recombinant mutants. **(B)** To estimate the temperature stabilities, reAuXyn11A and reAEXynM^C5T^ were incubated from 35 to 60°C and other recombinant xylanases from 45 to 80°C, respectively, for 1.0 hour. ■, reAuXyn11A; ▲, reAEXynM; □, reAEXynM^C5T^; △, reAEXynM^P9S^; ○, reAEXynM^H14N^.

The temperature optima of reAuXyn11A and reAEXynM were 50 and 70°C, respectively (Figure 4A), while their temperature stabilities were 45 and 75°C (Figure 4B). After incubation at 55°C for 1.0 hour, reAuXyn11A retained only 32.5% of its original activity, while reAEXynM retained nearly 100%. Even if incubated at 85°C, reAEXynM still retained 39.9% of its original activity.

**Figure 5:**
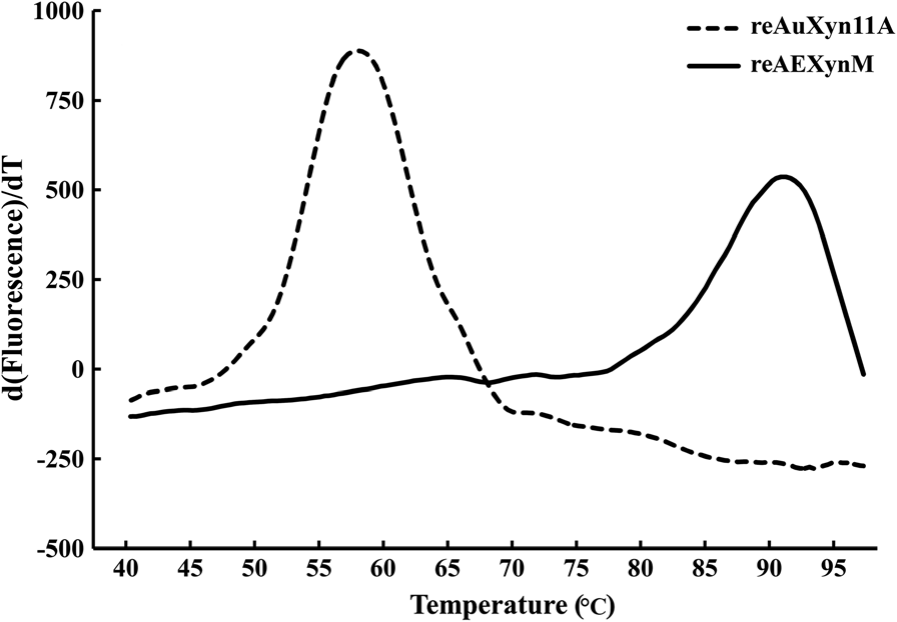
**Derivative melting curves of reAuXyn11A (dashed line) and reAEXynM (solid line).** The emission intensity of the fluorescence dye was recorded from 40 to 99°C at an elevated rate of 1°C min^-1^.

The emission intensity of the fluorescence dye combined with hydrophobic regions of a protein was gradually increased as the protein was unfolding at high temperatures [22]. Based on this mechanism, the melting temperature (*T*_m_) values of reAuXyn11A and reAEXynM were graphically determined from the derivative melting curve of 57.6 and 91.6°C, respectively (Figure 5), which corresponded well to their temperature stability levels (Figure 4B). As a result, by substituting the N-terminal 33 amino acids of AuXyn11A with the corresponding 38 ones of *Ev*Xyn11^TS^, the *T*_m_ value of reAEXynM increased by 34.0°C as compared with that of reAuXyn11A.

### Hydrolytic products from corncob xylan

**Figure 6:**
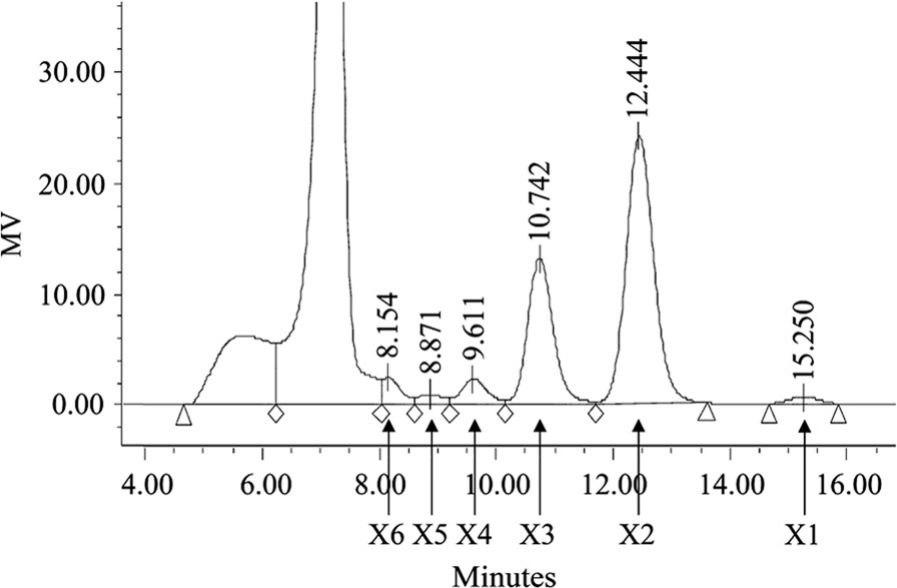
**HPLC analysis of the hydrolytic products released from corncob xylan by reAEXynM at pH 4.6 and 60°C for 3.0 hours.** The positions of xylose (X1), xylobiose (X2), xylotriose (X3), xylotetraose (X4), xylopentaose (X5), and xylohexaose (X6) are shown by arrows.

The hydrolytic products from corncob xylan by reAEXynM at different intervals were analyzed by HPLC. As the reaction time extended, the contents of xylobiose, xylotriose, xylotetraose, and xylopentaose increased, whereas that of xylohexaose decreased (Table 1). Only a trace of xylose was detected in the hydrolytic process, suggesting that reAEXynM is suitable for the production of XOSs. After incubation at pH 4.6 and 60°C for 3.0 hours, the contents of xylobiose and xylotriose as the major hydrolysates from corncob xylan were 5.081 and 2.492 mg mL^-1^, respectively (Figure 6). The hydrolytic conditions (except 40°C) and results of reAuXyn11A (Additional file 1) were similar to those of reAEXynM. It was reported that xylotriose as the major hydrolytic product was released from birchwood and wheat bran xylans by reBlxA with contents of 1.330 and 0.546 mg mL^-1^, respectively [23]. XOSs with low degrees of polymerization (DP = 2 to 6), produced from xylans by endoxylanases, have been proven to be able to promote proliferation of bifidobacteria, the beneficial microorganisms in the human intestine. Demand for this functional food additive has shown a rapid growth over the last two decades [24].

**Table 1:** Hydrolytic products released from corncob xylan by reAEXynM

**Incubation time (hours)**	**Content of the hydrolytic product (mg mL**^**-1**^**)**^**a**^					
	**Xylose**	**Xylobiose**	**Xylotriose**	**Xylotetraose**	**Xylopentaose**	**Xylohexaose**
0.5	0.033	0.780	1.635	0.414	0.161	0.510
1.0	0.051	1.932	2.234	0.432	0.166	0.362
2.0	0.079	2.621	2.366	0.442	0.172	0.353
3.0	0.097	5.081	2.492	0.443	0.174	0.344

^a^The contents of hydrolytic products were analyzed by HPLC.

### Determination of the crucial amino acids in the N-terminus

A total of 20 different amino acids between AEXynM and AuXyn11A were selected to identify the crucial amino acids that contribute to the high thermostability of AEXynM. After MD simulation processes, the total energy values of AEXynM and its 20 hypothetic single mutants were calculated, respectively (Table 2). As a result, three single mutants (AEXynM^C5T^, AEXynM^P9S^, and AEXynM^H14N^) with the highest total energy values were identified. Compared with AEXynM, three mutants displayed increases in total energy values from -11.66 to -10.96, -11.16, and -11.25 kJ mmol^-1^, respectively, which may contribute to their decreased thermostabilities [25]. The AEXynM^C5T^-, AEXynM^P9S^-, and AEXynM^H14N^-encoding genes were constructed as designed theoretically and expressed in *P. pastoris*, respectively. After flask expression tests, three recombinant strains, labeled as *P. pastoris* GSAEM^C5T^4-1, GSAEM^P9S^4-3, and GSAEM^H14N^4-5, were selected, expressing the highest reAEXynM^C5T^, reAEXynM^P9S^, and reAEXynM^H14N^ activities of 597.3, 609.2, and 558.8 U mL^-1^, respectively.

**Table 2:** Total energy values of AEXynM and its 20 hypothetic mutants

**Xylanase**	**Total energy value**	**Xylanase**	**Total energy value**
	**(kJ mmol**^**-1**^**)**		**(kJ mmol**^**-1**^**)**
AEXynM	-11.66		
AEXynM^C5T^	**-10.96**	AEXynM^P26G^	-11.40
AEXynM^L6S^	-11.35	AEXynM^T28D^	-11.53
AEXynM^S8P^	-11.46	AEXynM^N30T^	-11.31
AEXynM^P9S^	**-11.16**	AEXynM^F31Y^	-11.70
AEXynM^Q10S^	-11.55	AEXynM^C32T^	-11.53
AEXynM^F13E^	-11.43	AEXynM^L33N^	-11.75
AEXynM^H14N^	**-11.25**	AEXynM^L34G^	-11.32
AEXynM^F18Y^	-11.39	AEXynM^E35D^	-11.46
AEXynM^K23T^	-11.62	AEXynM^G36A^	-11.33
AEXynM^S25G^	-11.34	AEXynM^R38A^	-11.41

### Characterization of the recombinant mutants

The recombinant mutants, reAEXynM^C5T^, reAEXynM^P9S^, and reAEXynM^H14N^, displayed the same molecular mass (24.7 kDa) as reAEXynM on SDS-PAGE (Figure 3, lanes 5, 6, and 7). The specific activities of purified recombinant mutants were 10,146, 10,315, and 9,876 U/mg, respectively, slightly higher than that (9,529 U mg^-1^) of reAEXynM. The temperature optima of mutants were 55, 65, and 60°C, respectively (Figure 4A), lower than that (70°C) of reAEXynM. To evaluate the temperature stabilities, the residual activities of mutants were measured after incubation at various temperatures for 1.0 hour (Figure 4B). Both reAEXynM^P9S^ and reAEXynM^H14N^ were stable at 70°C, and reAEXynM^C5T^ at 55°C. After incubation at 70°C, reAEXynM^C5T^ lost all of its activity, while reAEXynM^P9S^ and reAEXynM^H14N^ retained 95 and 89% of their original activities, respectively. Moreover, reAEXynM^P9S^ and reAEXynM^H14N^ showed lower residual activities than reAEXynM after incubation at 80°C. These analytical results demonstrated that the three single mutations had negative effects on the thermostability of AEXynM as predicted by total energy value calculation, among which one single mutation of C5T caused the most significant decrease in thermostability. In other words, Cys^5^, Pro^9^, and His^14^ in the replaced N-terminus contributed mainly to the high thermostability of AEXynM.

### Analysis of the three-dimensional structure of AEXynM

**Figure 7:**
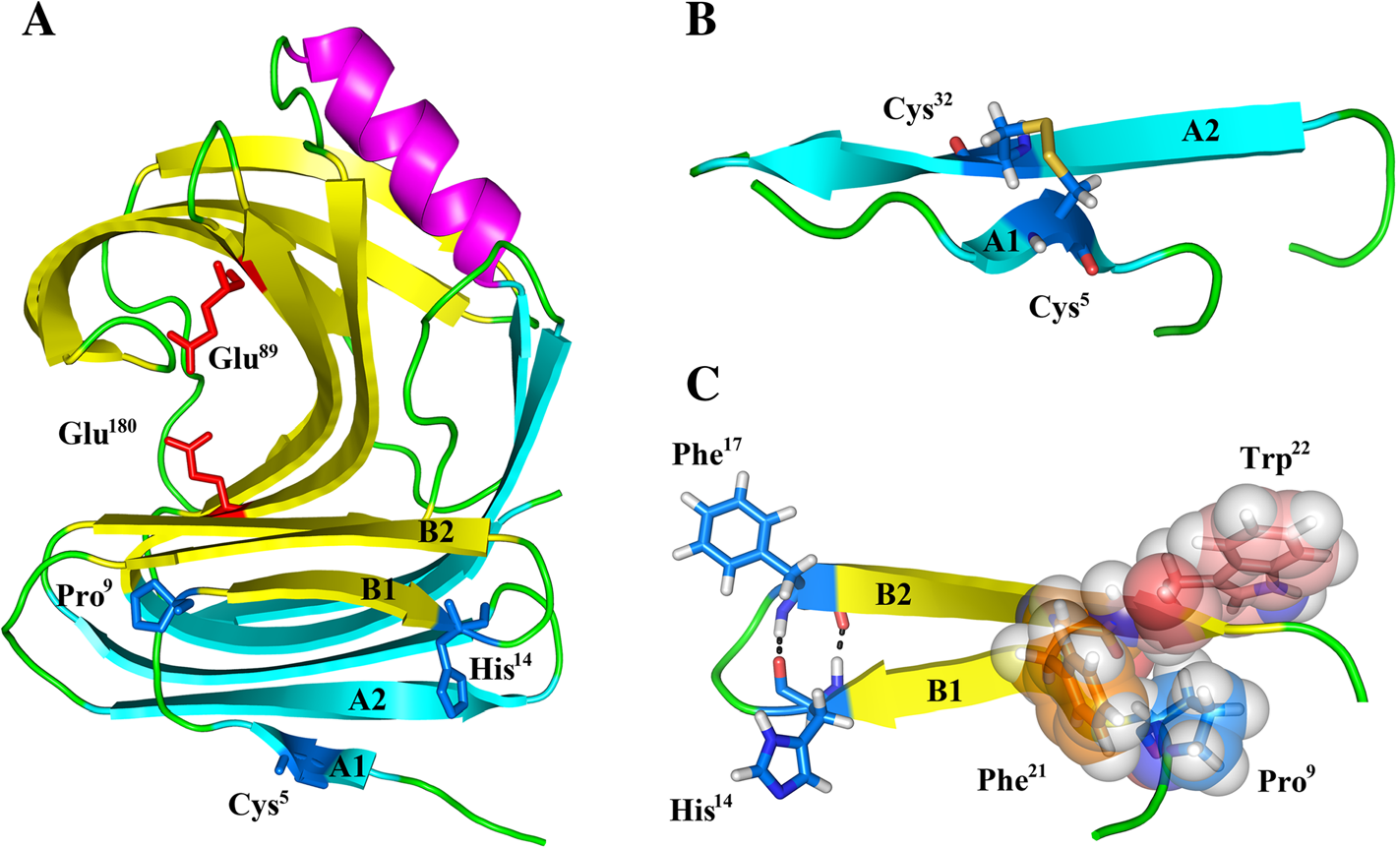
**Analysis of the three-dimensional structure of AEXynM. (A)** The three-dimensional structure of AEXynM predicted by MODELLER 9.9. Two invariant catalytic residues, Glu^89^ and Glu^180^, reside at the center of an active region. The amino acid residues (Cys^5^, Pro^9^, and His^14^) mainly responsible for the high thermostability of AEXynM are located in β-strands A1 and B1, respectively. **(B)** One disulfide bridge (Cys^5^-Cys^32^) is illustrated in the locally magnified three-dimensional structure. **(C)** The residues (Pro^9^, Phe^21^, and Trp^22^) represented with spheres compose a hydrophobic interaction cluster. The hydrogen bond between His^14^ and Phe^17^ is illustrated with a dashed line.

The three-dimensional structure of AEXynM conforms to the family 11 xylanase of the overall crystal structure, resembling a partially closed right hand (Figure 7A). It is composed mainly of one α-helix and 15 β-strands that are arranged in two mostly antiparallel β-sheets (β-sheet A and B). The invariant catalytic residues, Glu^89^ and Glu^180^, reside at the centre of an active region of AEXynM, where the β*-*1,4-D-xylosidic linkages of xylan insert and get cleaved.

The experimental results revealed that Cys^5^, Pro^9^, and His^14^ in the N-terminus were mainly responsible for the high thermostability of AEXynM. To elucidate the mechanism of thermostability, the intramolecular interactions relative to the three amino acid residues were analyzed using the Protein Interactions Calculator (PIC) server (Indian Institute of Science, Bangalore, India; http://pic.mbu.iisc.ernet.in) [26]. The results showed that a unique disulfide bridge (Cys^5^-Cys^32^), which cannot be present in AuXyn11A devoid of β-strand A1 (Figure 7B), may confer the enhanced thermostability on AEXynM by decreasing the entropy value of protein unfolding [27]. In this work, to experimentally verify the presence of the disulfide bridge in AEXynM, the purified reAEXynM was incubated with 5.0 mM (final concentration) dithiothreitol (DTT) in 20 mM Tris-HCl buffer (pH 8.0) at 40°C for 30 minutes, followed by measuring xylanase activity under standard assay conditions. As a result, the temperature optimum of reAEXynM treated with DTT decreased from 70 to 60°C. Considering that DTT reduces the disulfide bridge, the decrease in thermostability of reAEXynM after treatment with DTT demonstrated the presence of the disulfide bridge in AEXynM, which greatly contributed to its high thermostability. Some researchers also reported the effect of the disulfide bridge on the thermostability of xylanases [11,28].

The amino acid residue Pro^9^, located at the front of β-strand B1, was surrounded by two hydrophobic residues (Phe^21^ and Trp^22^) in the β-strand B2. The hydrophobic interactions of Pro^9^ with Phe^21^ and Trp^22^ as well as a hydrogen bond between His^14^ and Phe^17^ (Figure 7C) could stabilize the local configuration between β-strands B1 and B2 as a result of the high thermostability of AEXynM [10].

## Conclusions

The thermostability of a mesophilic AuXyn11A was clearly improved by substituting its N-terminal 33 amino acids with the corresponding 38 ones of a hyperthermostable *Ev*Xyn11^TS^. The temperature optimum, stability, and *T*_m_ value of reAEXynM were 70, 75, and 91.6°C, respectively, which were much higher than those of reAuXyn11A. Xylobiose and xylotriose as the major products were excised from corncob xylan by reAEXynM. In addition, it was demonstrated that Cys^5^, Pro^9^, and His^14^ in the replaced N-terminus contributed mainly to the high thermostability of AEXynM. The resulting reAEXynM can become a superior candidate for industrial processes at high temperatures, exemplified by preventing microbial contamination in the production of XOSs. This work will provide an effective strategy for improving the thermostabilities of other enzymes.

## Materials and methods

### Strains, vectors, and culture media

*A. usamii* E001, isolated from the soil in China as reported previously [14], was used as the donor of *Auxyn11A. Escherichia coli* JM109 and vector pUCm-T (Sangon, Shanghai, China) were applied for gene cloning, while *E. coli* DH5α and vector pPIC9K (Invitrogen) were used for construction of the recombinant expression vectors. *E. coli* JM109 and DH5α were cultured at 37°C in the Luria-Bertani medium consisting of 10 g L^-1^ tryptone, 5 g L^-1^ yeast extract, and 10 g L^-1^ NaCl, pH 7.2. *P. pastoris* GS115 and its transformant were cultured and induced at 30°C in the YPD, MD, geneticin G418 containing YPD, BMGY, and BMMY media, which were prepared as described in the manual of the Multi-Copy Pichia Expression Kit.

### Primers for PCR

A primer dT-PR (full name: Oligo dT-M13 Primer M4; TaKaRa, Dalian, China) was applied for reverse transcription of the first strand cDNA, from which a gene, *Auxyn11A,* coding for AuXyn11A was amplified with primers AuX-F and AuX-R. Primers SyX-F, SyX-R, and AuX-R were used for cloning of a hybrid xylanase AEXynM-encoding gene, *AExynM*. Three single mutant genes, *AExynM*^C5T^, *AExynM*^P9S^, and *AExynM*^H14N^, were constructed by PCR with the corresponding forward primers AM5-F, AM9-F, and AM14-F and a reverse primer AuX-R, respectively. As listed in Additional file 2, all PCR primers (except dT-PR) used in this work were synthesized by Sangon.

### Cloning of the genes *Auxyn11A* and *Syxyn11*

The gene *Auxyn11A* [GenBank: DQ302412] was amplified from the *A. usamii* total RNA extracted using the RNA Extraction Kit (Sangon) by RT-PCR. Meanwhile the gene *Syxyn11* [GenBank: JX459567], with optimized synonymous codons that bias towards *P. pastoris*, was artificially synthesized according to the *Ev*Xyn11^TS^-encoding gene sequence [GenBank: EU591743]. The two xylanase genes obtained were inserted into pUCm-T and then transformed into *E. coli* JM109, respectively, followed by DNA sequencing. Two resulting recombinant T-vectors containing the proper inserts, designated pUCm-T-*Auxyn11A* and pUCm-T-*Syxyn11*, were used as the parent genes for construction of a hybrid xylanase gene, *AExynM*.

### Computational prediction of the hybrid xylanase

The B-factor values, that is, atomic displacement parameters, of amino acid residues were generated by MD simulation towards the three-dimensional structure of a protein using the GROMACS 4.5 package (Royal Institute of Technology, Stockholm, and Uppsala University, Uppsala, Sweden; http://www.gromacs.org/), and then calculated using the B-FITTER program as described by Reetz *et al*. [29]. The RMSD value, which is an important index for evaluating the thermostability of a protein conformation, was defined as the C_α_-atomic displacement parameter of a protein from its original three-dimensional structure to the changed one at a high temperature and a certain time. The smaller the RMSD value of a protein, the smaller its C_α_-atomic displacement range, that is, the more thermostable its conformation [30].

In this work, the three-dimensional structure of AuXyn11A was homologically modeled by using the MODELLER 9.9 program (University of California San Francisco, San Francisco, CA, USA; http://salilab.org/modeller) based on the crystal structure of a hyperthermostable xylanase, *Ev*Xyn11^TS^, expressed in *E. coli* [PDB: 2VUL]. The B-factor values of AuXyn11A or *Ev*Xyn11^TS^ were calculated after a 15 ns MD simulation process at a temperature of 300 K. Based on the comparison of B-factor values between AuXyn11A and *Ev*Xyn11^TS^, a predicted hybrid xylanase, AEXynM, was designed by substituting a suitable N-terminal segment of AuXyn11A with the corresponding one of *Ev*Xyn11^TS^. To further evaluate the thermostability of AEXynM, the three-dimensional structures of three xylanases (AuXyn11A, AEXynM, and *Ev*Xyn11^TS^) were subjected to MD simulation processes at 500 K for 10 ns, respectively, followed by calculating their RMSD values using the g_rms software of the GROMACS 4.5 package.

### Construction of the hybrid xylanase gene

The AEXynM-encoding gene, *AExynM,* was constructed by substituting the 5′-end DNA fragment (99 bp in length) of *Auxyn11A* with the corresponding one (114 bp in length) of *Syxyn11* with the megaprimer PCR method [31]. Using pUCm-T-*Syxyn11* as the template, the DNA fragment (SyX) was amplified by first-round PCR with primers SyX-F (with an *Eco*RI site) and SyX-R at following conditions: denaturation at 94°C for 3 minutes; 30 cycles at 94°C for 30 seconds, 56°C for 30 seconds, 72°C for 15 seconds; and elongation at 72°C for 10 minutes. Then, the complete gene *AExynM* was amplified from pUCm-T-*Auxyn11A* by second-round PCR using fragment SyX and AuX-R (with a *Not*I site) as the primers under the same conditions as mentioned above, except an elongation at 72°C for 45 seconds in 30 cycles. The target PCR product was purified by using the EZ-10 Spin Column DNA Gel Extraction Kit (Bio Basic Canada Inc, Markham, ON, Canada) and inserted into pUCm-T, followed by transforming it into *E. coli* JM109. The resulting recombinant T-vector containing the gene *AExynM*, named pUCm-T-*AExynM*, was confirmed by DNA sequencing.

### Site-directed mutagenesis of AEXynM

For determining the crucial amino acids in the replaced N-terminus that contributed to the high thermostability of AEXynM, 20 hypothetic single mutant xylanases were designed by substituting the N-terminal of various amino acids of AEXynM with the corresponding ones of AuXyn11A (Figure 1B and Table 2), respectively. The three-dimensional structures of AEXynM and 20 hypothetic mutants were then homologically modeled and subjected to MD simulation processes at 300 K for 2 ns, respectively, followed by calculating their total energy values using the g_energy software of the GROMACS 4.5 package.

The total energy value of a protein was closely correlated with the entropy value of its unfolding [27]. It has been demonstrated that the lower the total energy value of a protein, the more thermostable its three-dimensional structure or conformation [25,27]. In this work, three hypothetic mutants (AEXynM^C5T^, AEXynM^P9S^, and AEXynM^H14N^), with the highest total energy values, were selected for site-directed mutagenesis. Their encoding genes (*AExynM*^C5T^, *AExynM*^P9S^, and *AExynM*^H14N^) were constructed by PCR, respectively. The target PCR products were gel-purified, inserted into pUCm-T, and transformed into *E. coli* JM109, respectively. The resulting recombinant T-vectors, named pUCm-T-*AExynM*^C5T^, pUCm-T-*AExynM*^P9S^, and pUCm-T-*AExynM*^H14N^, were confirmed by DNA sequencing.

### Expression of the xylanase genes

The *Auxyn11A*, *AExynM,* and three mutant genes were excised from five recombinant T-vectors by digestion with *Eco*RI and *Not*I, and inserted into pPIC9K digested with the same enzymes, followed by transforming them into *E. coli* DH5α, respectively. The resulting recombinant expression vectors were then linearized with *Sal*I, and transformed into *P. pastoris* GS115, respectively, by electroporation using Gene Pulser apparatus (Bio-Rad, Hercules, CA, USA) according to the manufacturer’s instruction.

All *P. pastoris* transformants were primarily screened based on their ability to grow on a MD plate, and successively inoculated on the geneticin G418-containing YPD plates at increasing concentrations of 1.0, 2.0, and 4.0 mg mL^-1^ for the screening of multiple copies of integrated xylanase genes, respectively. *P. pastoris* transformed with pPIC9K was used as the negative control (*P. pastoris* GSC). Expression of the xylanase gene in *P. pastoris* GS115 was performed according to the instruction of the Multi-Copy Pichia Expression Kit with slight modification [20].

### Purification of the expressed recombinant xylanases

After the *P. pastoris* transformant was induced by methanol for 72 hours, a total of 50 mL of cultured supernatant was brought to 45% saturation by adding solid ammonium sulfate, followed by centrifugation. Solid ammonium sulfate was then added to the supernatant up to 75% saturation. The resulting precipitate was harvested, dissolved in 4 mL of 20 mM Na_2_HPO_4_-NaH_2_PO_4_ buffer (pH 6.5), and dialyzed against the same buffer overnight. The dialysate was concentrated to 1 mL by ultrafiltration using a 10kDa cut-off membrane (Millipore, Billerica, MA, USA) and then loaded onto a Sephadex G-75 column (GE Healthcare, Little Chalfont, UK; inner diameter of 1.6 × 80 cm), followed by elution with the same buffer at a flow rate of 0.3 mL min^-1^. Aliquots of 1.5 mL eluent containing only a target xylanase were pooled and concentrated for further study. All purification steps were performed at 4°C unless stated otherwise.

### Enzyme activity and protein assays

Xylanase activity was assayed by measuring the amount of reducing sugars released from birchwood xylan (Sigma-Aldrich), using the 3,5-dinitrosalicylic acid (DNS) method [20]. One unit (U) of xylanase activity was defined as the amount of enzyme liberating 1 μmol of reducing sugar equivalent per minute under the assay conditions (at pH 4.6 and 50°C for 15 minutes). SDS-PAGE was performed according to the method of Laemmli [32] on a 12.5% gel. The isolated proteins were visualized by staining with Coomassie Brilliant Blue R-250 (Sigma-Aldrich). The protein concentration was measured with the BCA-200 Protein Assay Kit (Thermo Scientific, Waltham, MA, USA), using BSA as the standard.

### Temperature optima and stabilities

The temperature optima were measured, respectively, under the standard xylanase activity assay conditions, except temperatures ranging from 35 to 60°C for reAuXyn11A as well as from 45 to 80°C for reAEXynM and its single mutants (reAEXynM^C5T^, reAEXynM^P9S^, and reAEXynM^H14N^). To estimate the temperature stabilities, reAuXyn11A and reAEXynM^C5T^ were incubated in the absence of substrate at various temperatures (35 to 60°C) and other recombinant xylanases incubated at temperatures from 45 to 80°C, respectively, for 1.0 hour. The temperature stability in this work was defined as a temperature, at or below which the residual activity of xylanase, measured under the standard assay conditions, retained over 85% of its original activity.

### Measurement of the melting temperature

The *T*_m_ is defined as a temperature, at which half of a protein’s three-dimensional structure is unfolded as the temperature elevates. The higher the *T*_m_ value of a protein or enzyme, the more thermostable its three-dimensional structure [33]. The *T*_m_ value of xylanase in this work was measured using the Protein Thermal Shift (PTS) method and the PTS kit on ABI 7500 Real Time PCR apparatus (Applied Biosystems, Carlsbad, CA, USA) according to the manufacturer’s instruction. The purified xylanase was mixed with fluorescence dye and placed into a 96-well PCR plate, followed by heating from 40 to 99°C at an elevated rate of 1°C min^-1^. Deionized water instead of protein was used as the negative control. The excitation and emission wavelengths were 490 and 530 nm, respectively. Four replicates were performed independently. The *T*_m_ value of xylanase was considered as a temperature corresponding to the peak value in the derivative melting curve, which was plotted using the PTS software.

### Corncob xylan hydrolysis and HPLC analysis

Corncob xylan, prepared by using the alkali extraction method as reported previously [34], was suspended in 20 mM Na_2_HPO_4_ citric acid buffer (pH 4.6) at a concentration of 25 mg mL^-1^. A total of 40 mL of corncob xylan suspension was then incubated with reAEXynM (300 U g^-1^ xylan) at 60°C for different intervals. The hydrolytic reaction was terminated by boiling for 10 minutes. Hydrolytic products released from xylan by reAEXynM as well as the standard D-xylose, xylobiose, xylotriose, xylotetraose, xylopentaose, and xylohexaose (Megazyme, Wicklow, Ireland) were analyzed by HPLC, respectively. Isolation of xylose and XOSs was performed with a Sugar-PakI column (Waters, Milford, MA, USA; inner diameter of 6.5 × 300 mm), using pure water as the mobile phase at a flow rate of 0.5 mL min^-1^. The column temperature was kept at 85°C and the sample injection volume was 10 μL. Sugar peak areas were recorded using a Waters 2414 Refractive Index Detector (Waters). The content of each hydrolytic product was measured by quantifying its peak area with that of the corresponding standard D-xylose or XOS whose content was known.

## Abbreviations

BMGY: Buffered glycerol-complex medium; BMMY: Buffered methanol-complex medium; BSA: Bovine serum albumin; Cys: Cysteine; DNS: 3,5-Dinitrosalicylic acid; DP: Degree of polymerization; DTT: Dithiothreitol; EDTA: Ethylene diamine tetraacetic acid; GH: Glycoside hydrolase; His: Histidine; HPLC: High performance liquid chromatography; MD: Molecular dynamics; PCR: Polymerase chain reaction; PIC: Protein Interactions Calculator; Pro: Proline; PTS: Protein Thermal Shift; RMSD: Root mean square deviation; RT-PCR: Reverse transcription polymerase chain reaction; Tm: Melting temperature; XOS: xylooligosaccharide; YPD: Yeast extract peptone dextrose.

## Competing interests

The authors declare that they have no competing interests.

## Authors’ contributions

HZ carried out the computational prediction, cloning, and expression. JL helped to analyze the hydrolytic products and drafted the manuscript. JW and YY helped to purify and characterize the xylanases. MW directed the overall study and revised the manuscript. All authors read and approved the final manuscript.

## Supplementary Material

Additional file 1 **HPLC analysis of the hydrolytic products released from corncob xylan by reAuXyn11A at pH 4.6 and 40°C for 3.0 hours.** The positions of xylose (X1), xylobiose (X2), xylotriose (X3), xylotetraose (X4), xylopentaose (X5), and xylohexaose (X6) are shown by arrows. Click here for file

Additional file 2 PCR primers for construction of the xylanase genes. Click here for file
