# Supplementary material for: Determinants for the improved thermostability of a mesophilic family 11 xylanase predicted by computational methods
Source: Biotechnol Biofuels. 2014 Jan 6;7:3. doi: 10.1186/1754-6834-7-3 (PMC3895927; doi:10.1186/1754-6834-7-3)

Additional file 1. HPLC analysis of the hydrolytic products released from corncob xylan by reAuXyn11A at pH 4.6 and 40°C for 3.0 h. The positions of xylose (X1), xylobiose (X2), xylotriose (X3), xylotetraose (X4), xylopentaose (X5) and xylohexaose (X6) are shown by arrows.

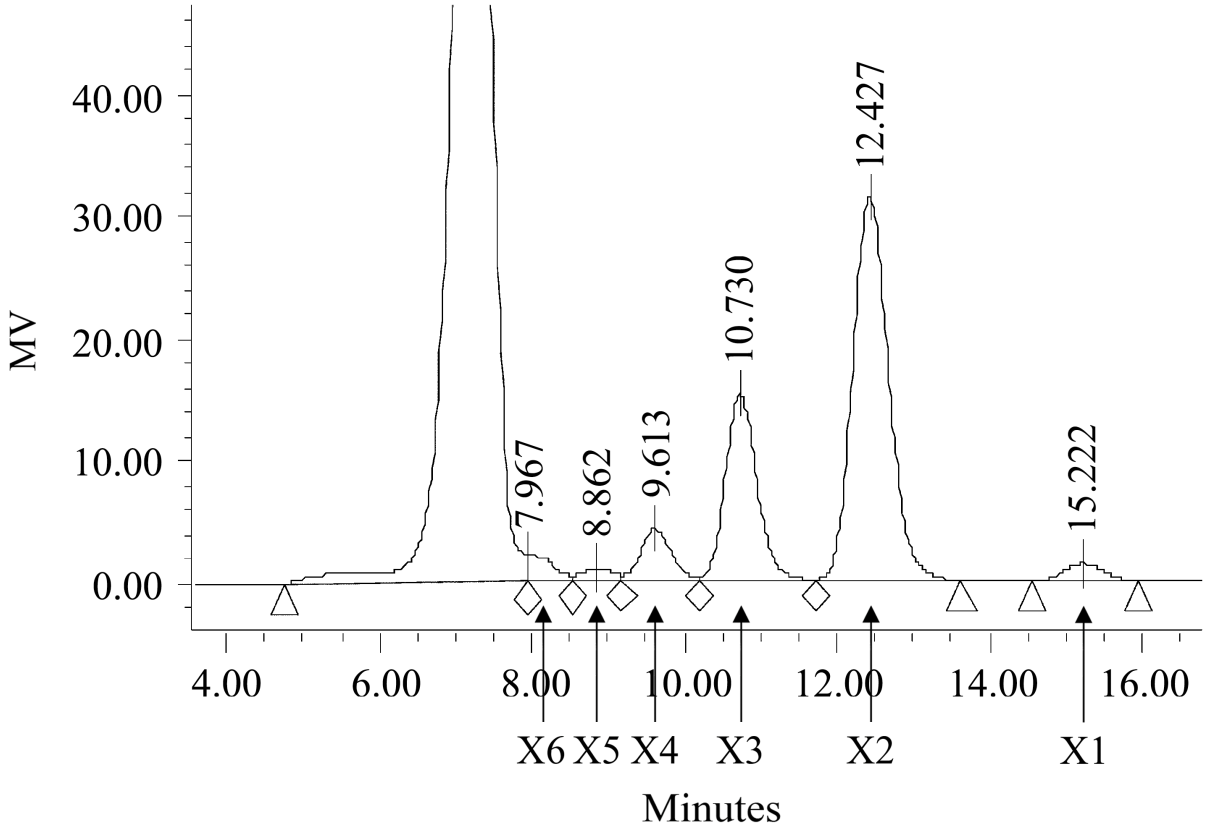

Supplement: Additional file 1 — HPLC analysis of the hydrolytic products released from corncob xylan by reAuXyn11A at pH 4.6 and 40°C for 3.0 hours. The positions of xylose (X1), xylobiose (X2), xylotriose (X3), xylotetraose (X4), xylopentaose (X5), and xylohexaose (X6) are shown by arrows. [file 1754-6834-7-3-S1.pdf]
