# Supplementary material for: Determinants for the improved thermostability of a mesophilic family 11 xylanase predicted by computational methods
Source: Biotechnol Biofuels. 2014 Jan 6;7:3. doi: 10.1186/1754-6834-7-3 (PMC3895927; doi:10.1186/1754-6834-7-3)
Supplement: Additional file 2 — PCR primers for construction of the xylanase genes. [file 1754-6834-7-3-S2.pdf]

Additional file 2. PCR primers for construction of the xylanase genes

Gene	Primer	Oligonucleotide sequence (5′–3′)[a]
Auxyn11A	dT-PR	GTTTTCCCAGTCACGAC-Oligo dT
	AuX-F	GAATTCGTTCCCCACGACTCTGTC
	AuX-R	GCGGCCGCTTACTGAACAGTGATGGACG
AExynM	SyX-F	GAATTCAACGCTCAAACTTGTCTTACC
	SyX-R	GGACCACTCAACAGTGTAACGACCACCCTCCA
	AuX-R	GCGGCCGCTTACTGAACAGTGATGGACG
AExynM^{C5T}	AM5-F	GAATTCAACGCTCAAACTactCTTACCTCTCCAC
AExynM^{P9S}	AM9-F	GAATTCAACGCTCAAACTTGTCTTACCTCTtcaCAAAC
AExynM^{H14N}	AM14-F	GAATTCAACGCTCAAACTTGTCTTACCTCTCCACAAA CTGGTTTTaacAA
	AuX-R	GCGGCCGCTTACTGAACAGTGATGGACG

[a] The underlined letters indicate *Eco*RⅠ and *Not*Ⅰ sites, respectively. All mutation sites in the forward primers are shown in lowercase letters.
